# Chronic thromboembolic pulmonary hypertension

**DOI:** 10.1007/s12471-014-0592-2

**Published:** 2014-08-29

**Authors:** B. E. Schölzel, R. J. Snijder, J. J. Mager, H W. van Es, H. W. M. Plokker, H. J. Reesink, W. J. Morshuis, M. C. Post

**Affiliations:** 1 Department of Cardiology, Amphia Hospital, Molengracht 21, 4818 CK Breda, the Netherlands; 2 Department of Cardiology, St. Antonius Hospital, Koekoekslaan 1, 3425 CM Nieuwegein, the Netherlands; 3 Department of Pulmonology, St. Antonius Hospital, Koekoekslaan 1, 3425 CM Nieuwegein, the Netherlands; 4 Department of Radiology, St. Antonius Hospital, Koekoekslaan 1, 3425 CM Nieuwegein, the Netherlands; 5 Department of Cardio-Thoracic Surgery, St. Antonius Hospital, Koekoekslaan 1, 3425 CM Nieuwegein, the Netherlands

**Keywords:** Pulmonary hypertension, Chronic thromboemboli, Pulmonary thromboendarterectomy, Pulmonary vasodilator therapy

## Abstract

Chronic pulmonary thromboembolic disease is an important cause of severe pulmonary hypertension, and as such is associated with significant morbidity and mortality. The prognosis of this condition reflects the degree of associated right ventricular dysfunction, with predictable mortality related to the severity of the underlying pulmonary hypertension. Left untreated, the prognosis is poor. Pulmonary endarterectomy is the treatment of choice to relieve pulmonary artery obstruction in patients with chronic thromboembolic pulmonary hypertension and has been remarkably successful. Advances in surgical techniques along with the introduction of pulmonary hypertension-specific medication provide therapeutic options for the majority of patients afflicted with the disease. However, a substantial number of patients are not candidates for pulmonary endarterectomy due to either distal pulmonary vascular obstruction or significant comorbidities. Therefore, careful selection of surgical candidates in expert centres is paramount. The current review focuses on the diagnostic approach to chronic thromboembolic pulmonary hypertension and the available surgical and medical therapeutic options.

**Electronic supplementary material:**

The online version of this article (doi:10.1007/s12471-014-0592-2) contains supplementary material, which is available to authorized users.

## Introduction

Chronic thromboembolic pulmonary hypertension (CTEPH) results from obstruction of the pulmonary vascular bed by non-resolving thromboemboli [1]. Although anatomic resolution of acute embolism is often incomplete, sufficient resolution occurs in the majority of patients to restore normal pulmonary haemodynamics associated with return to a pre-embolism functional status [2]. Several prospective studies have reported that between 0.6 and 4.6 % of acute pulmonary embolic survivors will develop symptomatic CTEPH [3, 4]. However, previous reports have shown that 25–63 % of patients with the established diagnosis of chronic thromboembolic disease do not have previously documented acute venous thromboembolism [5–7].

CTEPH is defined by the following observations after at least 3 months of effective anticoagulation: mean pulmonary artery pressure (PAP) >25 mmHg with a pulmonary capillary wedge pressure ≤ 5 mmHg; and perfusion defects detected by appropriate imaging techniques [8].

The incidence of acute pulmonary embolism is approximately 1:1000 [9], and diagnosed in about 16,000 patients annually in the Netherlands. The estimated cumulative incidence of CTEPH in the Netherlands is 0.5–1.5 % [3].

Historical data indicate that if left untreated CTEPH is associated with a poor five-year survival, ranging from 10–40 % depending on the pulmonary haemodynamics [10]. Pulmonary endarterectomy is the procedure of choice in symptomatic patients with surgically accessible CTEPH [1]. After surgery, most patients experience a substantial haemodynamic improvement, which is associated with improvements in functional status and long-term survival [1, 11]. The 30-day mortality after pulmonary endarterectomy ranges from less than 5 % in the most experienced centres to 10 % in others [12, 13]. However, in 30–50 % of patients pulmonary endarterectomy is not possible (inoperable CTEPH) due to either distal pulmonary vascular obstruction or significant comorbidities thought to be associated with unacceptably high risk [14].

Furthermore, approximately 10–15 % of operated patients suffer from persistent or recurrent pulmonary hypertension [15]. When pulmonary endarterectomy is not possible or when there is residual CTEPH after pulmonary endarterectomy, medical treatment with pulmonary hypertension-specific medication may be an option. Therefore, careful selection of the most appropriate treatment of CTEPH patients should be done in specialised centres.

## Pathophysiology and risk factors of CTEPH

Unlike pulmonary arterial hypertension (PAH), where vascular remodelling tends to occur in small pulmonary arteries, CTEPH is associated with prominent obstructions in larger vessels, combined with small vessel disease. The pathophysiology of CTEPH remains unclear. The embolic hypothesis suggests that CTEPH is the result of single or recurrent pulmonary embolism arising from sites of venous thrombosis [16]. The European CTEPH Registry has recently revealed that previous pulmonary embolism is detected in 74.8 % of all CTEPH patients while previous deep venous thrombosis is documented in 56.1 % of patients [17]. Recently, a prior history of splenectomy, ventriculo-atrial shunt for the treatment of hydrocephalus, thyroid replacement therapy, a history of malignancy and chronic inflammatory disorders such as osteomyelitis and inflammatory bowel disease, were associated with an increased risk of CTEPH [18]. Regarding details of the acute embolic event, systolic PAP greater than 50 mmHg at the time of diagnosis of acute embolism or at hospital discharge, previous pulmonary embolism, and a larger degree of pulmonary vascular obstruction at the time of acute pulmonary embolism diagnosis have been identified as risk factors for CTEPH [4, 19, 20].

According to current knowledge, CTEPH emerges as a ‘dual’ pulmonary vascular disorder with thrombosis inducing major vessel vascular remodelling, combined with pulmonary arteriopathy (small pulmonary vessel disease) as a consequence of non-occluded area over-perfusion [5, 21, 22]. Lung biopsy findings obtained at the time of pulmonary endarterectomy demonstrate pathohistological changes in the microvasculature, similar to those seen in other forms of small-vessel pulmonary hypertension, distal to both obstructed and non-obstructed central arteries [21]. The arteriopathy is considered to be the cause of the haemodynamic and symptomatic decline over time by contributing to the elevated pulmonary vascular resistance (PVR), thereby adversely affecting cardiac function and eventually leading to the progressive haemodynamic instability and increased mortality observed in patients with CTEPH [10].

Endothelin-1 (ET-1) is a potent endogenous vasoconstrictor and is considered to contribute to the increase in vascular tone and pulmonary vascular remodelling associated with pulmonary hypertension [23]. It was demonstrated that endothelial signalling pathway components are upregulated in CTEPH [24]. Moreover, ET-1 levels were shown to significantly decrease after successful pulmonary endarterectomy [25]. These observations indicate that ET-1 may play a role in the development of the secondary arteriopathy observed in CTEPH. Therefore, ET-1 has been considered a potential target for medical therapy in selected CTEPH patients [26].

## Clinical manifestations and diagnostic work-up

Early in the course of the disease, the clinical presentation of CTEPH can be subtle, which may contribute to the delay in diagnosis (honeymoon period). The common symptom in patients with CTEPH is exertional dyspnoea, the result of increased dead space ventilation as well as a limitation in cardiac output response to increased physiological demand [27]. Progression of the disease and further limitation of cardiac output may lead to signs of right heart failure, exertion-related presyncope, and chest pain that may be due to decreased right ventricular coronary flow related to increased right ventricular systolic pressure and mass [28].

Physical examination findings early in the course of the disease may be entirely unremarkable, thereby contributing to diagnostic delay. As disease progression occurs, findings consistent with pulmonary hypertension develop: prominence of the right ventricular impulse, a closely split second heart sound with accentuation of its pulmonary component, a right ventricular S4 gallop, and varying degrees of tricuspid regurgitation. With the onset of right ventricular failure, jugular venous distension, peripheral oedema, hepatomegaly, ascites, a right-sided S3, and a widened split of the second heart sound may be present.

Symptoms of disease are nonspecific and often attributed to other cardio-respiratory disorders, deconditioning, or even psychogenic disorders. Recent registry data showed a median time interval of 14.1 months between the first symptoms and CTEPH diagnosis [17]. Indeed, CTEPH is associated with a poor prognosis unless an early diagnosis is made and treatment is started [10].

Once the possibility of a pulmonary vascular disease has been considered, the diagnostic approach has three goals: first, to establish the presence and extent of pulmonary hypertension, second to determine its cause, and third to evaluate the therapeutic options.

Imaging studies are fundamental to decision-making with respect to diagnosis and operability of CTEPH. However, Klok et al. recently demonstrated that a simple diagnostic model based on ECG evaluation and NT-pro-BNP measurements was able to rule out CTEPH with a high level of confidence in patients with a documented history of acute and clinically suspected CTEPH [29].

Transthoracic echocardiography is sensitive for the detection of pulmonary hypertension and right ventricular dysfunction, but is not specific for the diagnosis of CTEPH. Common echocardiographic findings include right ventricular hypertrophy, dilatation and impaired right ventricular systolic function. Furthermore, right atrial enlargement, right ventricular pressure overload and tricuspid regurgitation can be found (video 1). However, echocardiography is not able to distinguish acute from sub-acute and chronic pulmonary embolism [30].

Stress echocardiography has been demonstrated to detect pulmonary vascular disease prior to a rise of PAP at rest. However, stress echocardiography needs further validation and therefore is not recommended as a routine method [31].

**Fig. 1:**
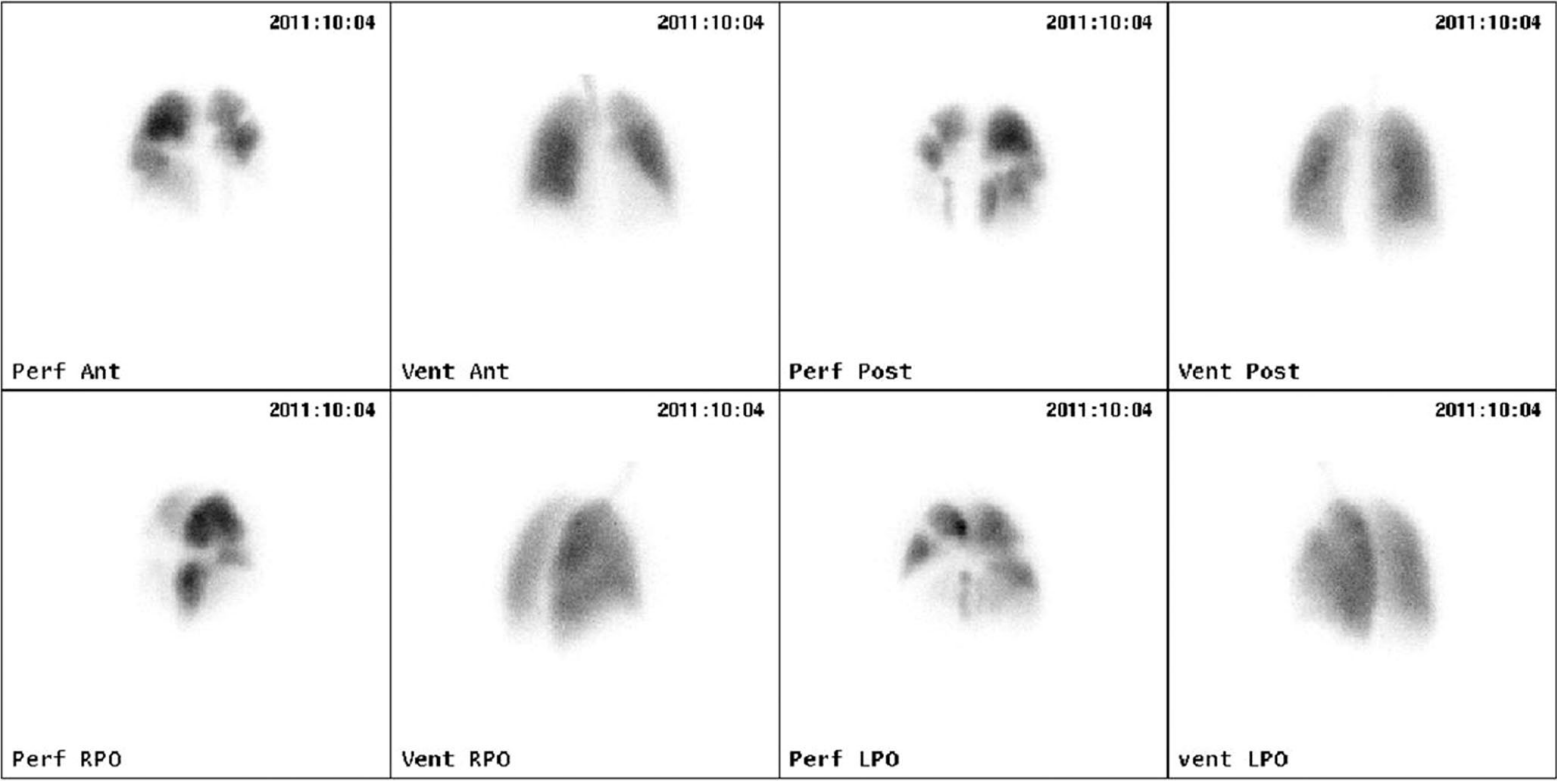
Lung perfusion scan in CTEPH, showing homogeneous ventilation and segmental defects in the perfusion scan

Ventilation-perfusion lung scanning should always be performed in the diagnostic work-up of pulmonary hypertension. In patients with CTEPH, the ventilation-perfusion scan invariably demonstrates one or more mismatched, segmental or larger defects (Fig. 1) [32]. Normal findings on ventilation-perfusion lung scanning rule out the diagnosis CTEPH and other investigations should be performed to find the cause of pulmonary hypertension [30]. Ventilation-perfusion lung scanning does not anatomically localise the extent of disease and cannot be used to determine surgical accessibility [30].

**Fig. 2:**
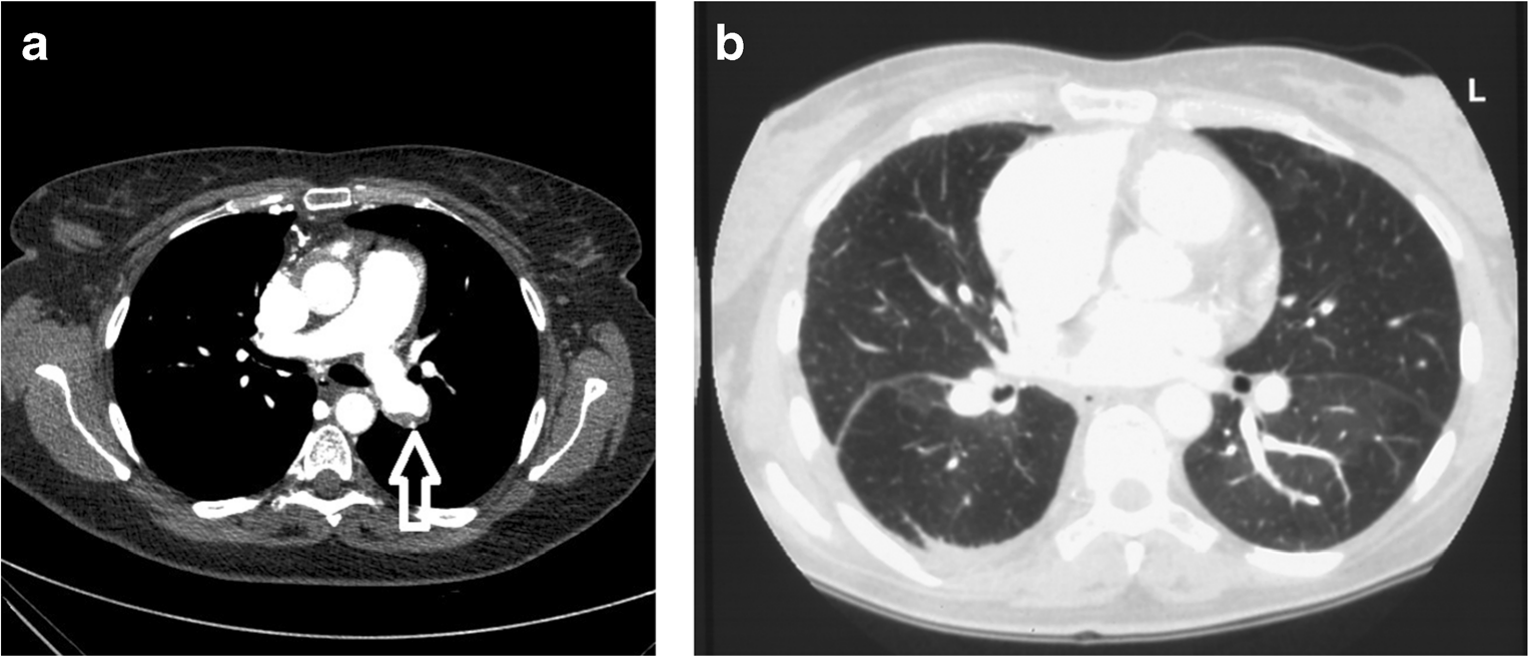
**a** Contrast-enhanced chest CT in CTEPH shows eccentric thrombotic material within the left pulmonary artery (*arrow*). **b** Contrast-enhanced chest CT scan in CTEPH before pulmonary endarterectomy showing mosaic perfusion

Computed tomography (CT) angiography may demonstrate a variety of parenchymal, vascular, or mediastinal abnormalities in patients with CTEPH. These include a mosaic parenchymal perfusion pattern, parenchymal scars, enlargement of the right ventricle and /or central pulmonary arteries, asymmetry in the size and distribution of lobar and segmental vessels, intraluminal thrombus, organised thrombus lining the pulmonary vascular walls, arterial webs or bands, and mediastinal collateral vessels (Fig. 2a and b) [33]. Accuracy of CT scanning has improved with technological advances, but a negative CT scan does not rule out CTEPH [34].

**Fig. 3:**
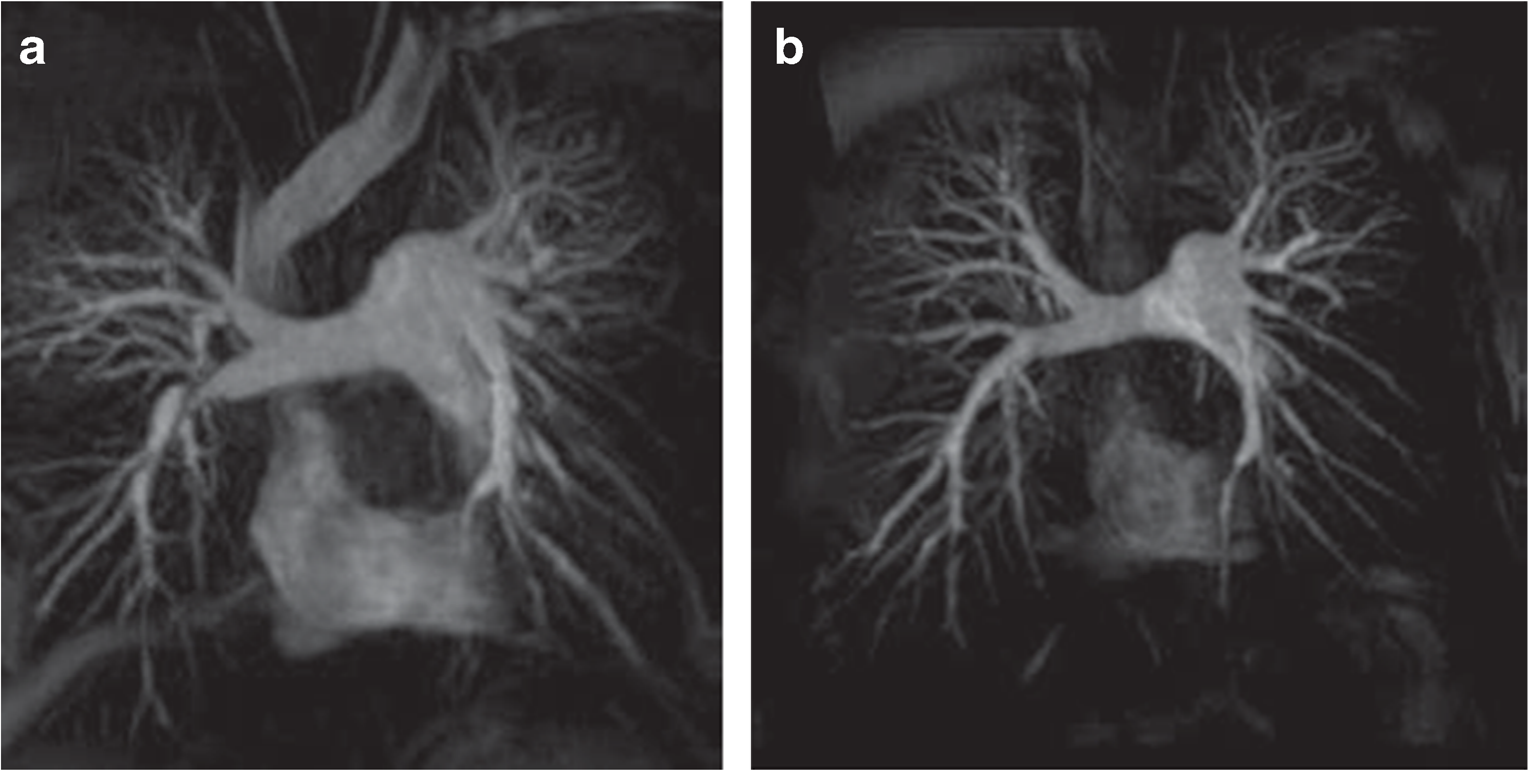
Magnetic resonance angiography in a patient with CTEPH before (**a**) and after (**b**) pulmonary endarterectomy showing a normalisation of the flow to the right lower lobe (*arrow*). Reproduced (Eur Respir Rev March 2012 21:32–39; doi:10.1183/09059180.00009211) with permission of the publisher

Magnetic resonance imaging is a non-invasive technique with no radiation exposure and offers great potential in CTEPH diagnosis and risk stratification before pulmonary endarterectomy [35]. It can be used for morphological, anatomical and functional assessment of both the heart and pulmonary circulation. Both high-resolution pulmonary angiography and dynamic temporally resolved angiography can be performed, with the latter enabling the detection of perfusion defects (Fig. 3) [36].

**Fig. 4:**
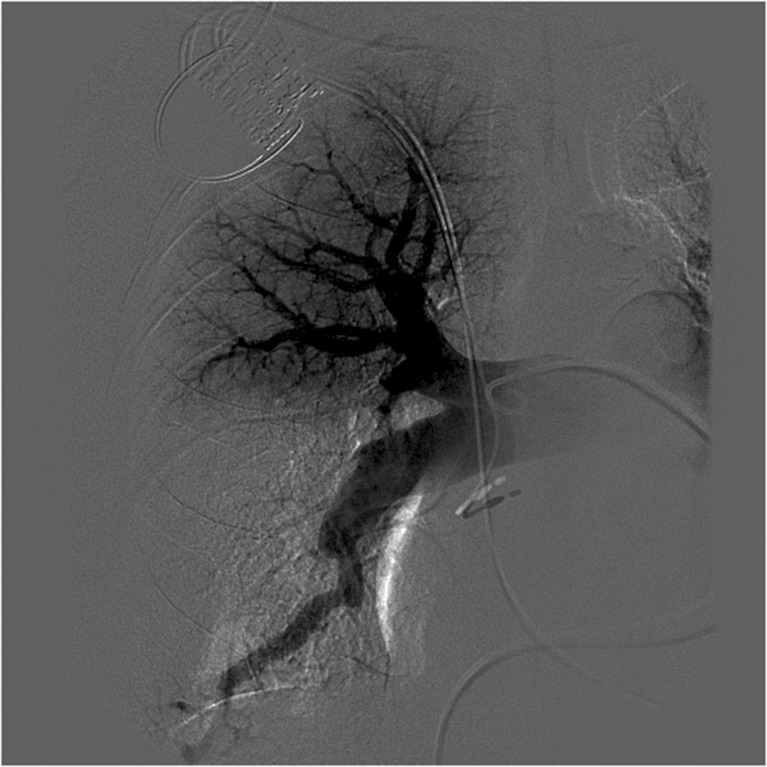
Pulmonary subtraction angiography of the right pulmonary artery showing a subsegmental branch occlusion of anterior trunk of the right upper lobe, an occlusion of the middle and lower lobe right pulmonary artery

Pulmonary angiography remains the gold standard. It establishes the diagnosis and assesses the operative resectability. Specific angiographic patterns that correlate with operative findings include pulmonary artery webs or bands, intimal irregularities, abrupt stenosis or pouches of major pulmonary arteries, and obstruction of lobar or segmental arteries at their origins (Fig. 4) [37].

All patients with suspected CTEPH should be referred to an experienced centre for confirmation of diagnosis and assessment of operability.

## Surgical treatment of CTEPH: pulmonary endarterectomy

**Fig. 5:**
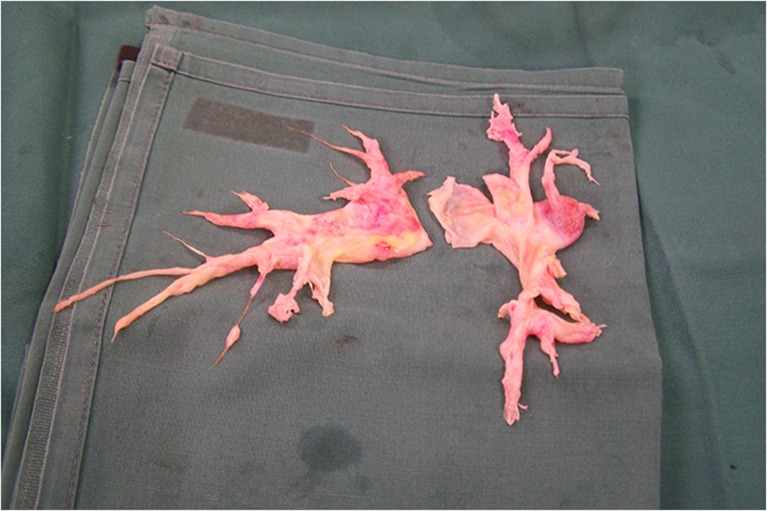
The endarterectomy specimen showing a pouch in the right lower lobe and the removal of fibrotic chronic thromboembolic material in the distal sub-segmental branches

The treatment of choice for symptomatic patients with CTEPH is pulmonary endarterectomy [1, 36, 38, 39]. The principle and aim of the operation is the removal of obstructive material with an immediate reduction in PVR (Fig. 5). The first successful true endarterectomy via sternotomy (with cardiopulmonary bypass standby) was performed in 1962 by Houk and colleagues [40]. Currently the pulmonary endarterectomy procedure involves median sternotomy, cardiopulmonary bypass and intermittent periods of hypothermic circulatory arrest to achieve a bloodless operative field and optimal exposure of the pulmonary artery [41, 42]. Periods of circulatory arrest are limited to 20-minute intervals. In general, an entire unilateral endarterectomy can usually be accomplished within this time by an experienced surgeon. After each period of circulatory arrest, reperfusion is carried out until at least 10 min has passed. The right pulmonary artery is incised where it passes the aorta to the division of the lower lobe arteries. On the left, the incision extends from the main pulmonary artery to the origin of the left upper-lobe branch [38]. Pulmonary thrombo-endarterectomy bears no resemblance to acute pulmonary embolectomy. The neo-intima in chronic thromboembolic disease is not easily recognisable as chronic thromboemboli and, thus, a true endarterectomy is necessary to restore pulmonary arterial patency. An endarterectomy plane is established between the intima and medial layer and so removing the fibrotic thromboembolic material. Considerable surgical experience with this procedure is required to identify the correct operative plane. A plane that is too deep will result in perforation of the vessel, while a plane that is too superficial will not result in an adequate endarterectomy.

Operability is based on the preoperative estimate of surgical classification [43] and the preoperative estimation of postoperative PVR, both of which determine risk of intervention and probable outcome.

### Outcome after pulmonary endarterectomy

Successful pulmonary endarterectomy markedly improves the haemodynamics, symptoms and functional status [12]. In the majority of patients undergoing pulmonary endarterectomy, both the short- and long-term haemodynamic outcomes are favourable and may be regarded as permanent [38]. A dramatic and immediate postoperative reduction of the mean PAP and PVR occurs. The mean reduction in PVR has approximated 70 % and a PVR in the range of 200 to 350 dyn·s·cm^−5^ can be achieved [15]. Patients in whom the postoperative PVR decreases by at least 50 %, to a value of less than 500 dyn·sec·cm^−5^, have a more favourable prognosis after surgery than those who do not: 30-day mortality rate of 1.2 % versus 5.7 % [41, 42]. The haemodynamic improvement is associated with an improvement in the symptoms and physical signs.

Overall 30-day mortality ranges from less than 5 % in the most experienced centres to 10 % in others [12–14]. A mortality rate of 1.3 % has been reported in patients at low risk based on their preoperative haemodynamic profile [38]. Potentially contributing to the improved outcome is a better understanding of the natural history of the disease, earlier and more selective surgical referral, improved diagnostic techniques, and advances in postoperative care [2].

Patients undergoing pulmonary endarterectomy are subject to many of the same postoperative complications as other cardiothoracic surgical procedures, such as atelectasis, pleural or pericardial effusion, diaphragmatic dysfunction and arrhythmias. However, reperfusion oedema and residual or persistent pulmonary hypertension are unique complications seen in the pulmonary endarterectomy patient and are associated with increased mortality [2].

Reperfusion oedema occurs in 10–40 % of patients, depending on the definition used. It is a high permeability oedema that occurs in regions that have been endarterectomised and reperfused [44]. Reperfusion oedema is an early postoperative complication with 60 % of cases presenting immediately after surgery, 30 % developing within the first 48 h postoperatively and the minority (10 %) occurring later during the hospitalisation (>48 h) [39]. The severity of preoperative pulmonary hypertension and the presence of residual pulmonary hypertension are associated with an increased risk of developing reperfusion pulmonary oedema [45].

Mortality due to CTEPH is low among patients who survive 3 months post-endarterectomy. Reported five-year survival rates vary between 72 and 93 % [12–14].

Residual or persistent pulmonary hypertension after pulmonary endarterectomy may result from incomplete endarterectomy, inaccessible chronic thromboemboli or small-vessel arteriopathy. The reported rates vary from 5 to 35 %, depending on the definition [46–48]. Although it is associated with a higher risk of late postoperative adverse events [12], functional improvement might be achieved and the survival seems to be equivalent to those patients without pulmonary hypertension after pulmonary endarterectomy [46]. This may indicate that the amount of PVR reduction matters, rather than the final PVR [2].

Residual pulmonary hypertension and reperfusion lung injury are often present in combination and, when severe, conventional therapy has been proved ineffective. Extracorporeal membrane oxygenation can be helpful as a supportive measure for patients with severe postendarterectomy complications and should be a standard of care in pulmonary endarterectomy centres [49].

Recently, clinical worsening has been used as a composite endpoint in pulmonary arterial hypertension trials, as described by McLaughlin [50]. It is a combination of mortality and different parameters which describe morbidity after the initiation of specific therapy for pulmonary hypertension. In recent reports, clinical worsening in CTEPH patients was defined as the combination of death, need for initiation of pulmonary hypertension-specific medication after pulmonary endarterectomy or a 15 % decrease in six-minute walking distance without improvement in New York Heart Association (NYHA) functional class. Clinical worsening has been described to occur in 20–30 % of the patients after pulmonary endarterectomy [51, 52].

### Predictors of outcome after pulmonary endarterectomy

After a haemodynamically successful pulmonary endarterectomy, the NYHA functional class improves and life expectancy increases [11]. Possible preoperative predictors for successful pulmonary endarterectomy are considered to be PVR, six-minute walking distance, radiological findings and comorbidities of the patients [1, 7, 14]. Several studies have associated high preoperative PVR (i.e. > 900–1100 dyn·s·cm^−5^) with increased mortality after pulmonary endarterectomy [14, 38, 53]. The postoperative PVR is also strongly related to mortality, and a decrease to less than 500 dyn·s·cm^−5^ has been reported as optimal [3]. Tscholl et al. demonstrated that age, right atrial pressure, NYHA functional class, cardiac output, creatinine and the number of angiographically involved segments were significant predictors for early death in univariate analysis. Age, right atrial pressure and female gender were identified as risk factors for unfavourable haemodynamic outcome after pulmonary endarterectomy [54]. Bonderman and associates demonstrated that the presence of associated medical conditions (i.e. splenectomy, inflammatory bowel disease and osteomyelitis) predicted increased operative risk and worse long-term outcome in CTEPH (i.e. higher mortality rates and more frequent occurrence of residual pulmonary hypertension) [47].

In a large prospective registry of Mayer et al., PVR three to 5 days after pulmonary endarterectomy and six-minute walking distance at diagnosis were identified as independent risk factors for in-hospital death [14]. Survivors had a higher six-minute walking distance and a lower PVR at diagnosis than non-survivors. The one-year mortality rate increased with increasing values of PVR at diagnosis to 12.8 % for those with a PVR exceeding 1200 dyn·s·cm^−5^ [14].

A recent report by Schölzel et al. demonstrated that the pulmonary artery diameter, indexed for body surface area, was able to predict the occurrence of mortality within 30 days after pulmonary endarterectomy and for the occurrence of clinical worsening during follow-up in patients with operable CTEPH [52].

## Medical treatment of chronic thromboembolic pulmonary hypertension

All patients with CTEPH should receive lifelong anticoagulation adjusted to a normalised target ratio between 2.0 and 3.0. The rationale is to prevent in situ pulmonary artery thrombosis and recurrent venous thromboembolism.

Consideration about commencing medical therapy for CTEPH patients should only occur following surgical assessment, as the currently available drugs are not alternatives to pulmonary endarterectomy [39].

Primary medical therapy and pretreatment with medical therapy prior to pulmonary endarterectomy in patients who appear to have surgically accessible chronic thromboembolic disease that seems proportionate with the degree of pulmonary hypertension are currently not recommended by the international guidelines. Patients with ‘out of proportion’ elevated PVR prior to surgery, persisting/residual pulmonary hypertension following pulmonary endarterectomy (due to distal obstructive thrombotic lesions situated beyond the sub-segmental level but also due to arteriopathy), and inoperable CTEPH are often considered for management with pulmonary hypertension -targeted therapies, despite the fact that these medications are not approved for the treatment of CTEPH [26, 55].

A substantial number of patients (with operable and inoperable CTEPH) are currently being treated off-label. In the International CTEPH Registry, 38 % of all patients were treated with at least one drug targeting pulmonary hypertension at diagnosis [17]. Most of the studies investigating the use of pulmonary hypertension-targeted therapies in the management of patients with distal CTEPH show beneficial effects [56, 57]. The BENEFiT study is a large randomised controlled trial that was performed in patients with inoperable CTEPH (*n* = 157) [26]. This study demonstrated a positive treatment effect of bosentan (an endothelin receptor antagonist) on haemodynamics in this patient population without improvement of exercise capacity [26].

In the recent CHEST-1 study, the efficacy and side-effect profile of riociguat (soluble guanylate cyclase stimulators) was evaluated in patients with inoperable CTEPH and patients with persistent or recurrent pulmonary hypertension after pulmonary endarterectomy [58]. Riociguat significantly improved exercise capacity and PVR in patients with CTEPH compared with placebo [58].

Selected patients with a predicted higher risk for postoperative mortality may benefit from preoperative medical treatment, especially those in NYHA functional class IV, those with a mean PAP greater than 50 mmHg, cardiac index less than 2 L·min^−1^·m^−2^ and/or PVR greater than 1200 dyn·s·cm^−5^ or signs of right heart failure [57, 59]. Whether improving pulmonary haemodynamics with preoperative pulmonary hypertension treatment also improves surgical outcome is unknown and remains largely speculative [60]. In a retrospective analysis of 9 patients who were treated with continuous intravenous epoprostenol (a prostacyclin analogue) before surgery, Bresser et al. found substantial improvements in cardiac index, mean PAP and total pulmonary resistance in all patients after pulmonary endarterectomy. However, impact on post-pulmonary endarterectomy morbidity and mortality could not be established [59]. In a prospective randomised study by Reesink et al., pulmonary haemodynamics and functional capacity were analysed in 25 pulmonary endarterectomy candidates treated with or without bosentan [61]. After treatment of 16 weeks, significant improvements were observed in mean PAP, total pulmonary resistance and six-minute walking distance in the bosentan group compared with controls. However, the outcome after pulmonary endarterectomy was similar in both groups [61].

Jensen et al. retrospectively analysed the medical treatment of the CTEPH patients referred to their centre for pulmonary endarterectomy [62]. Although the use of pulmonary hypertension-specific medication before surgery had significantly increased, there was no significant improvement in preoperative pulmonary haemodynamics and postoperative outcome [62].

The increased use of medications in operable patients could possibly delay referral of patients for pulmonary endarterectomy. Selection of suitable candidates for bridging therapy should be carefully carried out in expert centres .

## Conclusion

CTEPH has a poor prognosis if left untreated. Pulmonary endarterectomy is the treatment of choice, offering a potential cure. Peroperative mortality rates and postoperative outcome have improved the last decades. However, a substantial number of patients are not candidates for pulmonary endarterectomy due to either distal pulmonary vascular obstruction or significant comorbidities. Therefore, careful selection of surgical candidates in expert centres is paramount.

New developments in medical treatment of inoperable CTEPH and persistent or recurrent pulmonary hypertension after pulmonary endarterectomy show promising results. However, larger studies with mid- to long-term follow-up are needed.

## Electronic supplementary material

Below is the link to the electronic supplementary material.

Video 1 Transthoracic echocardiogram. Parasternal short axis at the level of the papillary muscles. There is systolic and diastolic flattening of the interventricular septum which suggests pressure and volume overload of the right ventricle. (AVI 17,846 kb)
